# Implementation of a hepatitis-themed virtual escape room in pharmacy education: A pilot study

**DOI:** 10.1007/s10639-023-11745-1

**Published:** 2023-04-05

**Authors:** Sunanthiny Krishnan, Ali Qais Blebil, Juman Abdulelah Dujaili, Sara Chuang, Angelina Lim

**Affiliations:** 1 School of Pharmacy, Monash University Malaysia, Bandar Sunway, Kuala Lumpur, Selangor 47500 Malaysia; 2 Faculty of Pharmacy and Pharmaceutical Sciences, Monash University, Parkville, VIC 3052 Australia; 3 Department of Pharmacy, Al-Rafidain University College, Baghdad, 10001 Iraq; 4 Swansea University Medical School, Swansea University, Wales, SA2 8PP UK

**Keywords:** Pharmacy education, Virtual escape room, Gamification, Educational game, Educational technology

## Abstract

As we enter a world of blended learning in higher education, an increased need for adaptation of teaching strategies to enhance engagement has been recognised to amplify learning outcomes online. Gamification has been identified as a creative tool to engage the current cohort of learners who are also characteristically tech-savvy. To this end, escape room games have gained considerable traction in medical and pharmacy education to promote learning, critical thinking and teamwork. In this pilot study we describe the implementation of a 60-minute, web-based hepatitis-themed escape room game within a Year 3 Pharmacotherapy unit at Monash University. A total of 418 students participated in this activity. Students’ knowledge gain on the topic was assessed through a pre- and post-intervention assessment, whereby a statistically significant improvement was seen in the knowledge score following implementation of the gaming activity (58.66% pre-intervention vs. 72.05% post-intervention, *p* < 0.05). The innovative learning activity was also well perceived by the students. Virtual escape room game is a viable pedagogical approach to teach and reinforce clinical concepts among pharmacy students. With the evolving landscape of education and learner demographics, investment in technology- enhanced game-based learning is a promising trajectory to support students’ growth in a learner-centered environment. A comparison between virtual escape room game and traditional teaching will further inform effectiveness of the gamification on long term knowledge retention.

## Introduction

### Gamification in education

Gamification has been gaining momentum in medical and health science education around the world, with studies showing that educational gaming stimulates students’ engagement and persistence on tasks, which in turn enhances deep learning (Cain & Piascik, 2015; Carvalho et al., 2015; Kafai & Burke, 2015). Broadly defined, gamification refers to the use of typical elements of game mechanics (e.g. storylines, avatars and rewards) in non-game context such as in an academic setting to enhance active engagement, motivation and thus maximise learning (Deterding et al., 2011; Faiella & Ricciardi, 2015). It has been credited as a useful form of active learning in education (Oestreich & Guy, 2022), affording an immersive experience for students by breaking the monotony of traditional didactic classroom environment.

The term gamification is often used interchangeably with “educational games”, “game-based learning” and “serious games” (Sera & Wheeler, 2017). The American Association of Colleges of Pharmacy (AACP) Academic Affairs Committee strongly promotes the development of educational games in pharmacy education to strengthen students’ acquisition of knowledge and professional development (Cain et al., 2014). Presentation of complex concepts of higher education in a gamified context motivates learners to review and integrate course-based knowledge through a creative yet meaningful medium of learning (Guckian et al., 2020b). A systematic review by Lumsden et al. (2016) amalgamates the fidelity of educational games on cognition which includes improved problem-solving skills, faster processing speed, being more goal-oriented and reaching a focused state of mind. Judicious and appropriate use of game-based pedagogy also creates social learning environments that support collaborations, competitions and interactions among students (Abu-Dawood, 2016).

### Educational escape room game

In recent years, there is a growing interest in Educational Escape Rooms, a novel form of serious games within pharmacy courses. An escape room game is a live-action team-based mission where participants collectively discover clues, solve puzzles and complete tasks in order to “escape” within a limited amount of time (Rosenkrantz et al., 2019). In the pharmacy education space, escape rooms have been designed around particular themes such as diabetes (Eukel et al., 2017), cancer (Wilby & Kremer, 2020) and heart failure (Plakogiannis et al., 2020), to name a few.

The unique, fast-paced and engaging architecture of escape room game has been well received in pharmacy education and perceived by students to positively impact their teamwork, engagement, problem-solving and clinical knowledge (Caldas et al., 2019; Eukel et al., 2017; Kavanaugh et al., 2020; Plakogiannis et al., 2020; Wilby & Kremer, 2020).

### Digital game-based learning

While game-based learning is frequently described in pharmacy education, the uptake of digital games is sparce and is largely varied in the level of technology used to deliver the exercise (Oestreich & Guy, 2022). A well-designed digital game can simulate realistic scenarios and enhance player’s immersive experience, potentially translating to a meaningful engagement. Although there are arguments that female students can be at a disadvantage if digital games were widely introduced in science, technology, engineering and mathematics (STEM) education, emerging studies are proving otherwise (Joiner et al., 2011).

Digital game-based learning has a natural appeal to the current generation of learners (Guckian et al., 2020) who are characteristically tech-savvy with a strong propensity for active-learning models. These “digital natives” (Autry & Berge, 2011), are engaged more effectively through technology-based gaming activities which align well with their generational preference for instant gratification, achievement recognition and team orientation (Jain & Dutta, 2019).

The current body of literature in pharmacy curricula predominantly discusses designs of escape room games implemented physically, whilst reports of digital escape rooms are limited. Badr (2022) describes a virtual geriatric escape room game designed using Google® forms for fifth-year pharmacy students. A gender-specific difference in students’ perception of the activity was reported while the impact of the activity on learning remains to be seen.

Identifying this research gap, a team of academics at School of Pharmacy, Monash University developed an innovative educational escape room on a digital platform. This article will focus on the implementation of a hepatitis-themed virtual escape room game in a pharmacy undergraduate programme.

## Materials and Methods

### Virtual escape room game design (Hepatitiscape©)

In this pilot study, a 60-minute, virtual educational escape room game based on hepatitis (*Hepatitiscape©)* was developed and implemented within a Year 3 Pharmacotherapy unit in the course. The web-based game consisted of three rooms and ten puzzles for students to work through as a team. Each team consisted of five to six members, with one member assuming the role of the ‘*player’*. The ‘player’ essentially had the navigational control within the gaming environment while working with the other members to solve the various puzzles. The goal of the game was to successfully escape the virtual building within 60 min.

A wide range of puzzle types were utilised to enhance students’ engagement and to elicit teamwork throughout the game (Table 1). The puzzles were built in sequential logic, whereby students would need to solve the first puzzle in order to advance to the next. To maintain this linearity, clues or passwords to unlock the subsequent puzzles were embedded in the preceding ones, such that failing to solve the puzzle would halt progression within the game.

**Table 1:** Hepatitis Escape Room Puzzles

Learning Objective (LO)			Gaming Task
Room 1	Puzzle 1	Demonstrate understanding of the genetic composition of Hepatitis B virus (HBV)	Jigsaw puzzle scavenger hunt Decoding message
	Puzzle 2	Demonstrate understanding of the risk factors for HBV infection	Word connect
	Puzzle 3	Demonstrate ability to describe hepatitis B vaccination schedule for patients on haemodialysis	Rebus puzzle
Room 2	Puzzle 4	Demonstrate ability to interpret hepatitis B serological markers	Math riddle Interpretive puzzle
	Puzzle 5	Demonstrate ability to describe travel vaccination against HBV	Archery game
Room 3	Puzzle 6	Familiarisation of the signs and symptoms of HBV infection	Sliding tiles puzzle
	Puzzle 7	Demonstrate ability to describe the clinical presentations of acute HBV infection	Word jumble
	Puzzle 8	Demonstrate ability to distinguish the clinical features of the various phases of chronic HBV infection	Drag & drop game
	Puzzle 9	Demonstrate ability to clinically evaluate and recommend appropriate management for a patient with HBV infection	Phone message response
	Puzzle 10	Identify goals of therapy in the management of chronic HBV infection	Flight simulation game

### Hepatitiscape© software development process

*Hepatitiscape*© is a single-user, three-dimensional (3D) interactive escape room game developed using Unity® game engine and rendered on WebGL® application programming interface. It is compatible to be played on desktops and/or laptops using Firefox® as the preferred browser. The game is fully playable with keyboard and mouse. The mouse is used to navigate and control the game, whilst the arrow keys or WASD keys are used to move around in the 3D gaming environment. The keyboard is also used to enter codes and passwords to unlock puzzles. Background music was added to the build for a more immersive experience during the play.

**Fig. 1:**
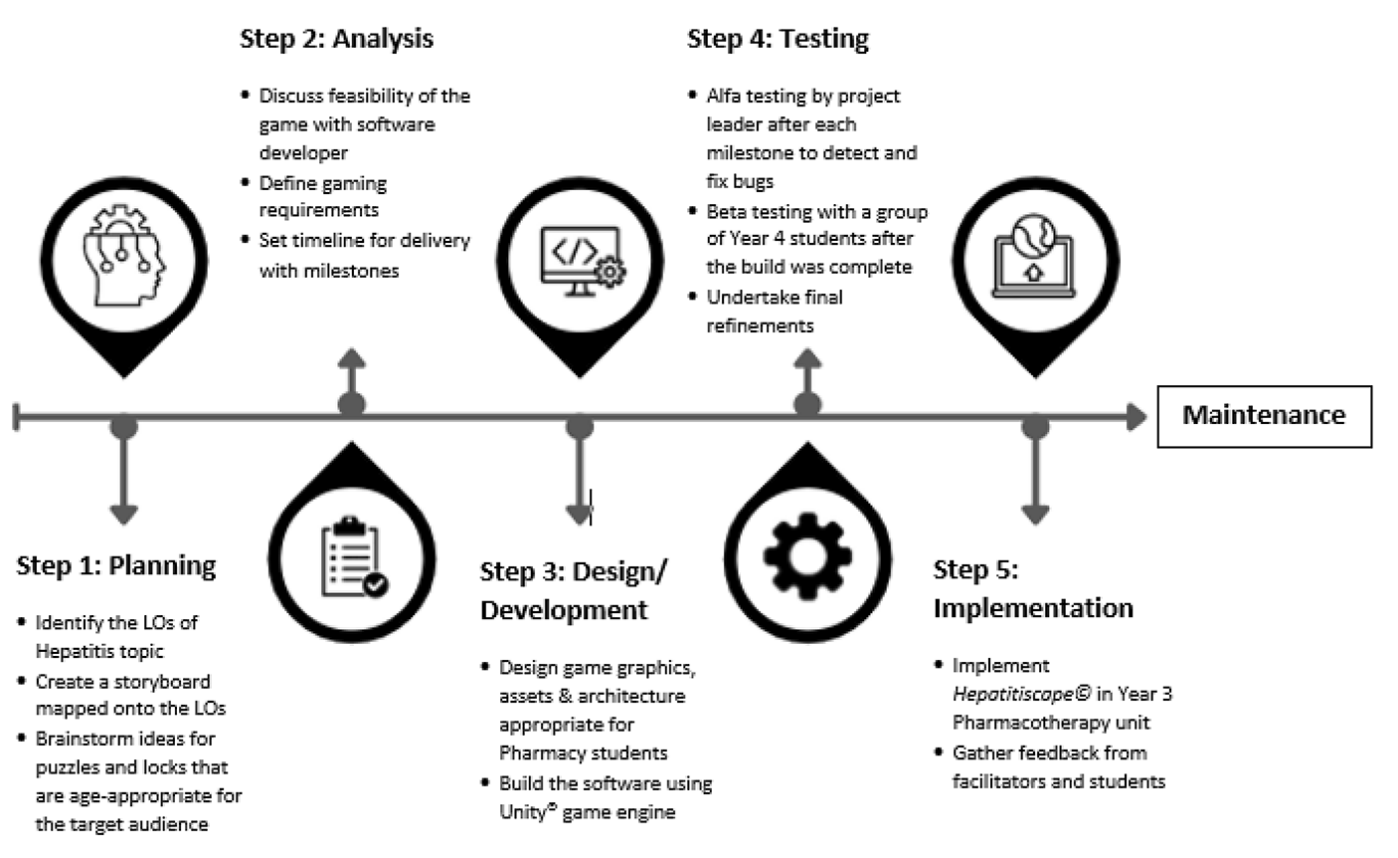
*Hepatitiscape©* Software Development Process

Figure 1 depicts the various stages of the gaming software development. The entire process required 6 months to complete.

The total cost of the project was USD 1200, which was largely allocated to the software developer’s and 3D modeler’s salary. After the initial software development, beta testing was undertaken with a group of Year 4 students to evaluate the playability of the game online and to gather feedback on the difficulty level of the puzzle designs. Following this process, several amendments were implemented to the design to make it more user-friendly and engaging.

### Implementation of Hepatitiscape©

The integrated curriculum of Bachelor of Pharmacy (Hons) programme at Monash University follows a prescribed format of topic delivery known as the DEAR teaching model (Forrester et al., 2021). On day 1 of the week, students indulge in self-directed pre-reading and related tasks (Discover) provided on the university’s learning management system (LMS). This is followed by an engaging interactive lecture (Explore) that emphasises the learning outcomes of the topic the next day. Students then participate in workshops to consolidate their knowledge on the topic on day 3 (Apply). The learning process is concluded on the fourth day with a close-the-loop lecture that revisits the core learning objectives and addresses any gaps in students’ understanding of the topic (Reflect).

Hepatitis is delivered in semester 1 of Year 3 as part of a 12-credit point therapeutic unit that covers various infectious diseases. *Hepatitiscape©* was conceptualised to replace the conventional workshop for the topic of hepatitis B, complementing three hours of prior learning on the topic. All the other elements of the topic delivery remained the same.

Prior to the gaming activity, students were supplemented with written instructions on how to play the virtual escape room game as well as a step-by-step guide on setting up their computers so as to be compatible with the online gaming platform. These were given as pre-workshop tasks made available to students on the LMS at the start of the hepatitis topic week so that students had ample time to set up their devices and to be familiar with the gameplay.

On the day of escape room game implementation, facilitators were engaged to help troubleshoot any technical issues that students may encounter during the gameplay. In the event students were unable to solve a puzzle, they were able to request for a hint from the facilitators. All facilitators were given a set of standard hints for each puzzle to give the students should they request for one. They were, however, strictly prohibited from giving students direct answers to any of the puzzles, so as not to hamper students’ critical thinking and problem-solving skills. Students were allowed to access their usual reference materials during the gameplay similar to their regular workshop settings.

The game-based learning activity was implemented simultaneously at both Malaysian and Australian campuses.

### ***Pre- and post- intervention assessment***

A set of ten multiple choice questions related to the topic of hepatitis B was administered to students immediately prior to the implementation of the virtual escape room game to assess their pre-intervention knowledge of the topic. Students were given ten minutes to complete the assessment and use of any resources was strictly prohibited during the attempt.

The same set of questions were administered again immediately after completion of the escape room game to measure their post- intervention knowledge gain, with ten minutes to complete without any references to any resources.

The assessment was built on SurveyMonkey® and administered online. Students were not informed of the pre-intervention assessment prior to attending the workshop, to prevent them from excessive preparation for the test which could potentially dilute the efficacy of the intervention. Similarly, they were not aware of the post-intervention test or that it was the same set of questions as the pre-intervention assessment.

### Statistical analysis

Data analysis was performed using SPSS version 26 (SPSS Inc., Chicago, IL). A Shapiro-Wilk’s test (p < 0.05) (Razali & Wah, 2011) and a visual inspection of histograms, normal Q-Q plots and box plots showed both pre-intervention and post-intervention assessment scores were not normally distributed, with a skewness of -0.210 (SE = 0.124) and a kurtosis of -0.657 (SE = 0.247) for the pre-intervention scores and a skewness of -0.615 (SE = 0.133) and a kurtosis of 0.078 (SE = 0.265) for the post-intervention scores. Mann-Whitney U Test was used to compare the scores between the pre-intervention and post-intervention assessment. Kruskal-Wallis test was used to compare the scores within pre-intervention and post-intervention groups.

## Results

### Pre- and post- intervention Assessment

A total of 418 students (132 and 286 students from Malaysian and Australian campuses, respectively) participated in the virtual escape room game activity that was conducted in May 2022. Out of this cohort, 388 students completed the pre-intervention assessment (92.8% response rate) and 337 students completed the post-intervention assessment, corresponding to a response rate of 80.6%. Demographics of the respondents are shown in Table 2.

**Table 2:** Demographics of Respondents

	Pre-Intervention		Post-Intervention	
	N (388)	Percentage (%)	N (337)	Percentage (%)
Monash University Campus Malaysia Australia	128 260	33.0 67.0	131 206	38.9 61.1
**Gender** Male Female Prefer not to mention	105 274 9	27.1 70.6 2.3	84 243 10	24.9 72.1 3.0
**Previous Work Experience** (*not including experiential placements*) Hospital/ Hospital Pharmacy Community Pharmacy None	19 203 166	4.9 52.3 42.8	19 179 139	5.6 53.1 41.2

A statistically significant improvement was noted in students’ knowledge assessment score following the implementation of the game-based activity. The average score increased by 13.39% (*p* < 0.05) from 58.66%, after participating in *Hepatitiscape©* (Table 3).

**Table 3:** Pre- and Post- Intervention Scores

	N	Average score (%)	Median score (%)	IQR^#^ (%)	*p*-value
**Pre-intervention**	388	58.66	60	20	**< 0.05**
**Post-intervention**	337	72.05	70	20	

^***#***^ ***IQR*** *– interquartile range*

The difference in the knowledge scores across sub-groups are populated in Table 4. All gender categories achieved higher scores after the game with statistical significance. Between-group analysis showed that all groups achieved similar scores before and after the gamification (*p* = 0.352 and *p* = 0.108 pre-intervention and post-intervention, respectively). Students’ previous work experience in a healthcare setting did not affect their performance in the assessment either before or after the gamification.

**Table 4:** Comparison of scores across sub-groups

	Pre- intervention		Post- intervention		*p*-value^c^
	Average score (%)	*p*-value^a^	Average score (%)	*p*-value^b^	
Gender					
Male	57.71	0.352	71.91	0.108	**< 0.05**
Female	58.76		71.69		**< 0.05**
Prefer not to mention	66.67		82.00		**0.047**
**Previous Work Experience**					
Hospital/ Hospital Pharmacy	63.68	0.429	77.37	0.103	**0.01**
Community Pharmacy	57.83		70.00		**< 0.05**
None	59.10		73.96		**< 0.05**

^a^ Comparison of scores between groups (pre-intervention)

^b^ Comparison of scores between groups (post-intervention)

^c^ Comparison of scores pre- and post-intervention

Beyond the immediate pre-and post-intervention assessment, students’ performance in the end-of-semester examination on the topic of hepatitis was also analysed. Students scored an average of 73.51% (N = 417) for this topic in the year 2022 compared to an average score of 70% (N = 364) in 2021.

### Student feedback

Following the implementation of *Hepatitiscape©*, feedback from students was sought through an online survey. A total of 105 students responded to the survey on their experience and perception of the virtual educational game (Table 5).

**Table 5:** Students’ experience and perception of *Hepatitiscape©*

Statements	Students’ Response (%)		
	**Strongly Disagree/ Disagree**	**Neutral**	**Strongly Agree/ Agree**
I have a better understanding of the hepatitis topic after this activity	0.95	1.90	97.15
The activity improved my critical thinking skills	0.95	3.81	95.24
The activity improved my problem-solving skills	0.95	4.76	94.29
The activity reinforced and helped me review my current knowledge (e.g., concepts and principles) about the hepatitis topic	0.95	0.95	98.10
The escape room activity was an effective way to learn new information related to hepatitis	0.95	1.90	97.15
The activity allowed more engagement in learning the topic	0.00	1.90	98.10
This activity improved our teamwork	0.00	0.95	99.05
I would recommend the use of game - based learning to students in other programmes	0.00	3.81	96.19
Overall, I enjoyed this activity	0.00	1.90	98.10

## Discussion

*Hepatitiscape©* was successful in increasing students’ immediate retention of knowledge on hepatitis concepts with a statistically significant 13.39% increase in average score on the post- intervention assessment. The results were consistent across genders, irrespective of their prior work exposure in hospitals or community pharmacies. Students who did not have any part time work experience in healthcare sectors demonstrated significant improvement in their knowledge on the topic, similar to their peers who have previous work experience, corroborating the fidelity of the game-based learning activity in augmenting students’ knowledge gain.

Serious games such as escape rooms are an entertaining and engaging method of delivering learning materials and have become a popular learning tool in medical (Backhouse & Malik, 2019; Guckian et al., 2020; Wiemker, 2015) and pharmacy education (Badr, 2022; Cain, 2019; Caldas et al., 2019; Clauson et al., 2019; Cole & Ruble, 2021; Richter & Frenzel, 2021; Eukel et al., 2017; Gordon et al., 2019; Kavanaugh et al., 2020) due to the generational shift in the current learner population and the societal impact of gamification (Guckian et al., 2020b). A good escape room activity allows for achievable goals, autonomy for freedom of choice for learners and incorporates teamwork (Guckian et al., 2020a). These elements are particularly meaningful for the current generation of learners who are naturally inclined to valuing choice and variety in their learning, as well as having a preference for collaborative social experiences (Jain & Dutta, 2019; Wilson & Gerber, 2008). Aside from these features, the project team feels that the novelty of virtual rendering of *Hepatitiscape©* also added to the appeal of the game among the students.

The pedagogic benefits of collaborative game-based virtual activities are attributable to the constructivist learning theory, which centres on the notion that people learn by personal discovery and problem-solving and that knowledge is constructed based on experiences (Whitton & Hollins, 2008). Virtual escape room games such as *Hepatitiscape©* provide opportunities for students to explore and navigate learning on their own in an environment that is built with a purposeful context for critical- thinking and problem- solving. Collaboration with others during this process enables students to work to their strengths and validate their ideas (Palloff & Pratt, 2003). Interestingly, our very own student feedback that was gathered after the implementation of *Hepatitiscape©* corroborates these notions whereby a vast majority of students reported the virtual game enhanced their critical thinking and problem-solving skills. Furthermore, a particularly high number of students responded that the online game improved their teamwork.

The online nature of *Hepatitiscape©* is also an asset for blended learning approach owing to its convenience of delivery. *Hepatitiscape©* is perpetually available online for students to play and practice any time, beyond the teaching hours. The single-player design of the game gives students the flexibility to either play individually or with their peers for their own practice outside of the workshop setting. As the programme is self-sufficient, it can run on its own without any facilitation. This eliminates the need for physical space and the costs associated with manpower, as well as the logistics of repeated set-ups of physical escape room games, which makes digital games a more sustainable educational tool in the long run. The virtual activity also affords unlimited attempts by the players which will reinforce the learning outcomes of the topic.

Comparison of end-of-semester examination results between the present cohort of students who participated in *Hepatitiscape©* with those who went through the regular workshop in 2021, revealed a similar average score on the hepatitis-related question, i.e., 73.51% and 70% in year 2022 and 2021, respectively. This suggests that digital gamification is equally as good as the traditional method of teaching and learning, although more robust study designs are warranted to validate this comparison. More research is also called for to investigate the benefits of virtual game-based activity on long term knowledge retention.

## Conclusion

In this pilot study, a hepatitis-themed virtual escape room game was successfully developed and implemented in a Year 3 Pharmacy undergraduate programme. The digital game-based learning activity was found to increase students’ knowledge gain on the topic of hepatitis. Students also found the virtual educational game engaging and enjoyable. They perceived the activity to be an effective way to learn clinical concepts in hepatitis as it reinforced their current knowledge on the topic. The escape room game improved their teamwork, critical thinking as well as problem-solving skills. Technology-enhanced educational gamification such as a virtual escape room is a viable pedagogical approach for pharmacy educators to explore, more so now as the global education landscape is restructured post-COVID-19 pandemic.

## Data Availability

The datasets generated during and/or analysed during the current study are available from the corresponding author on reasonable request.
